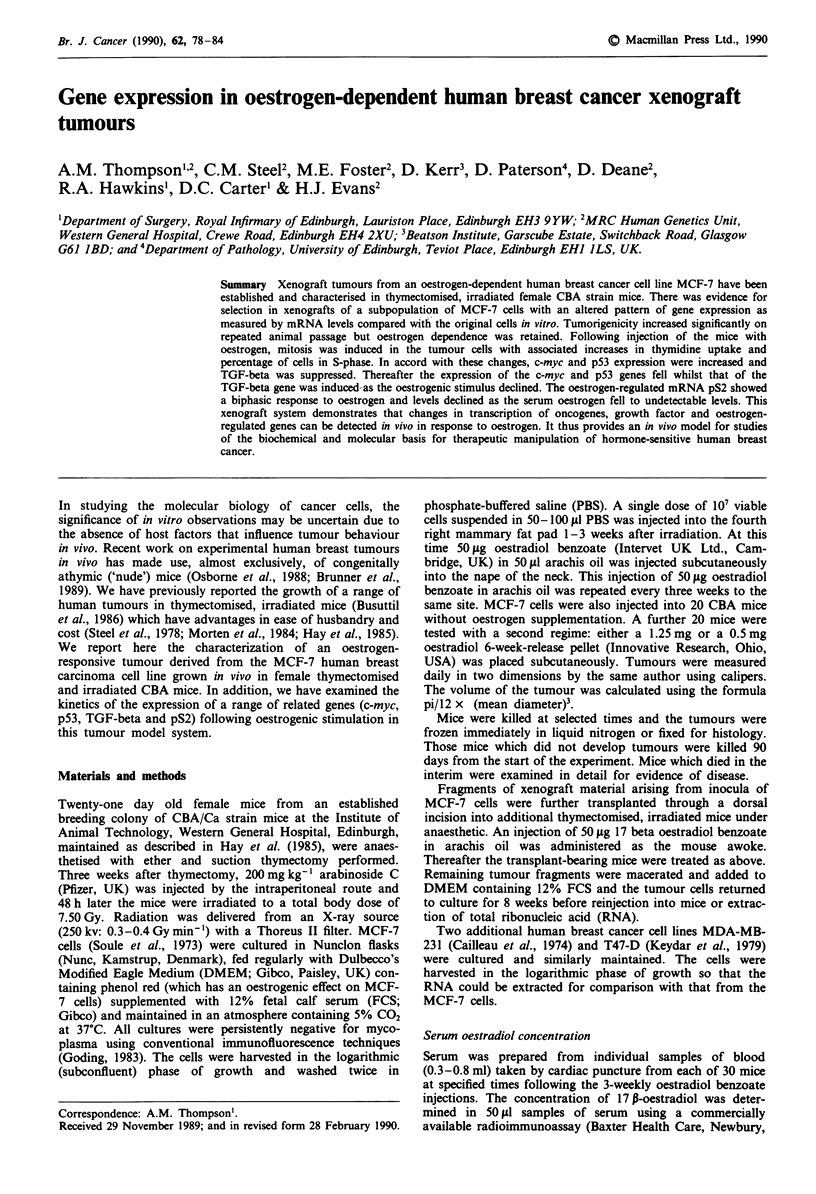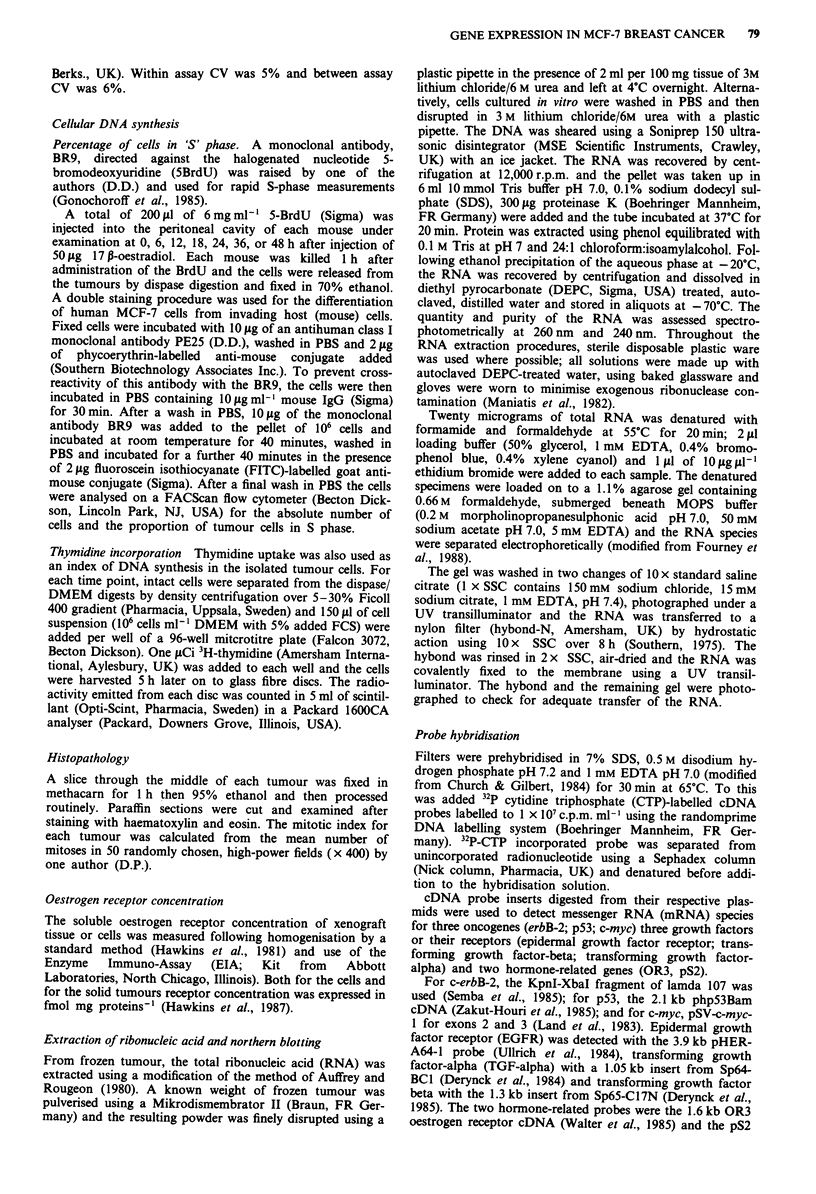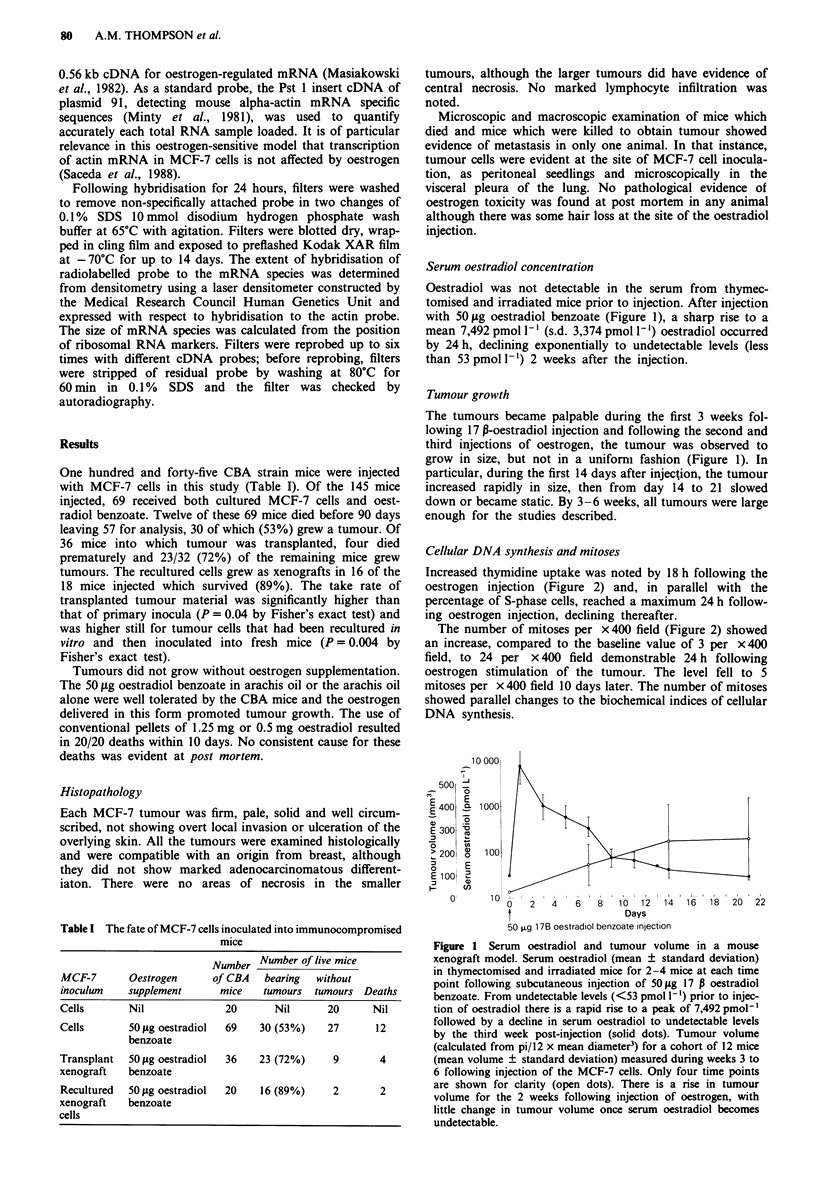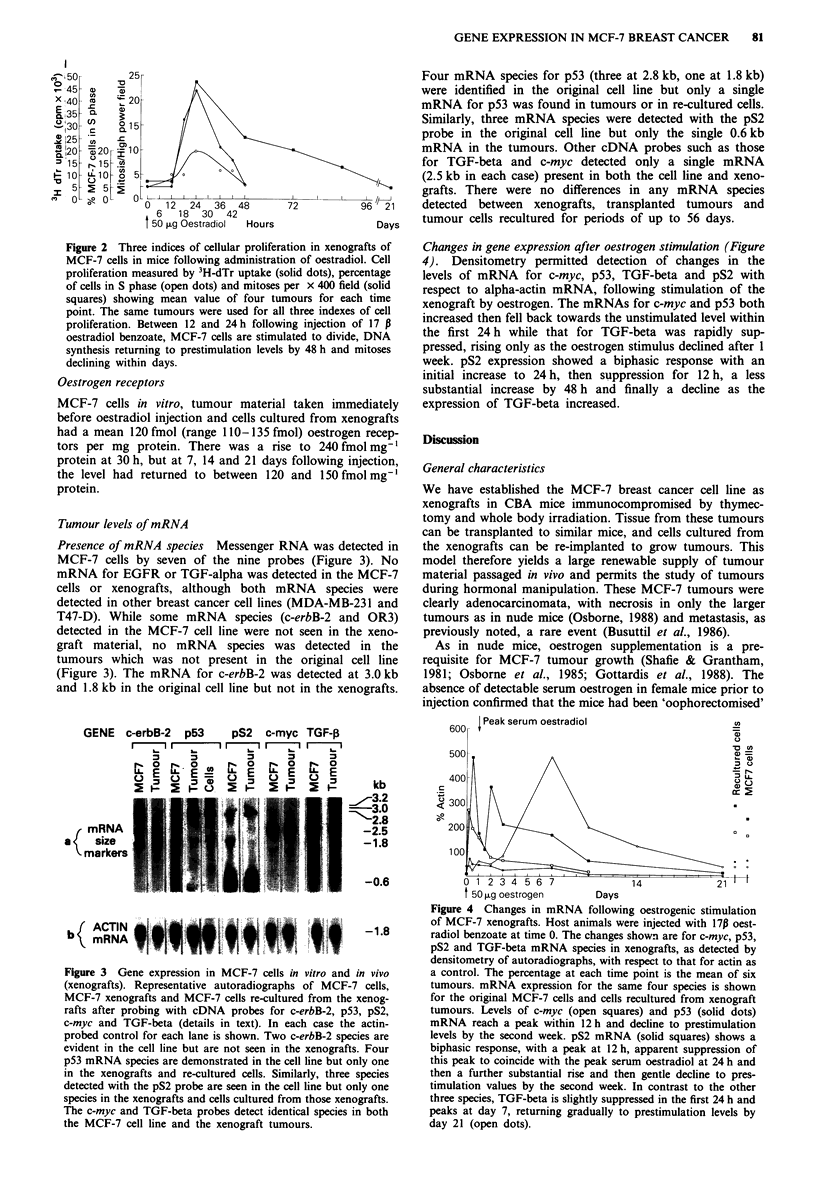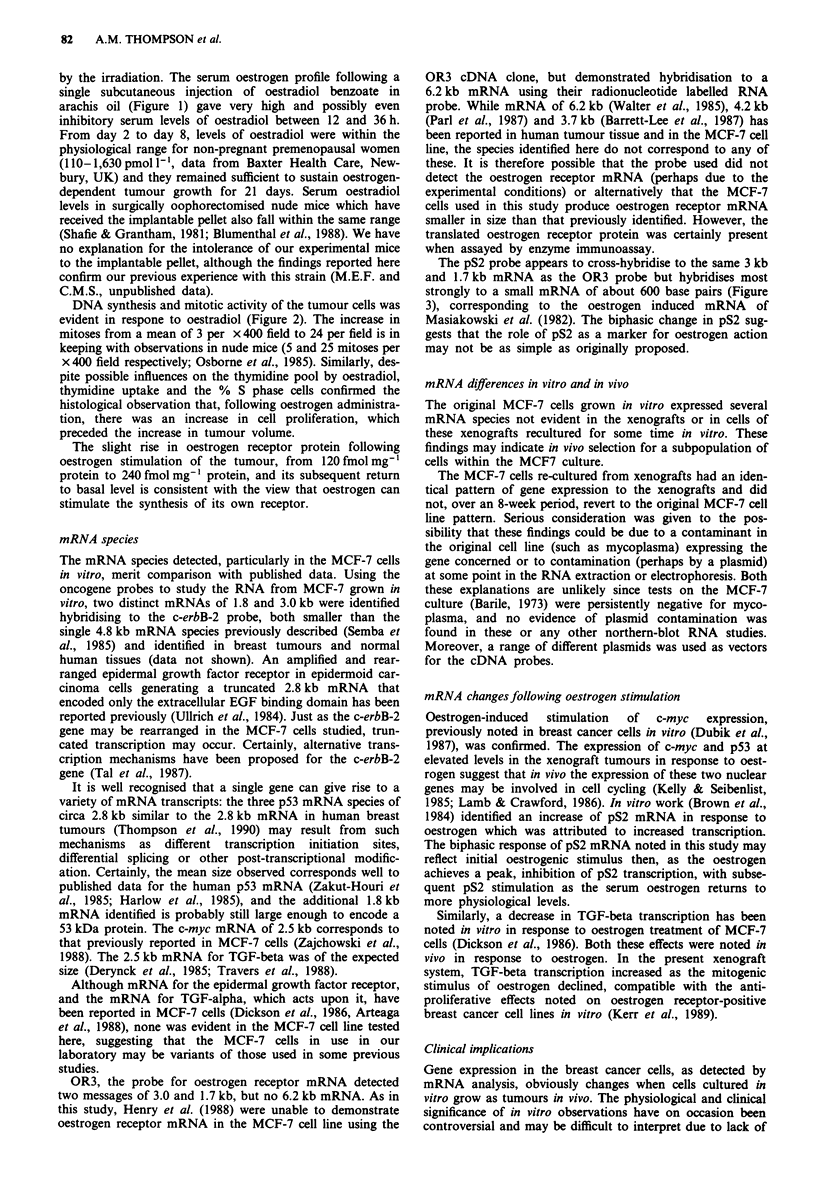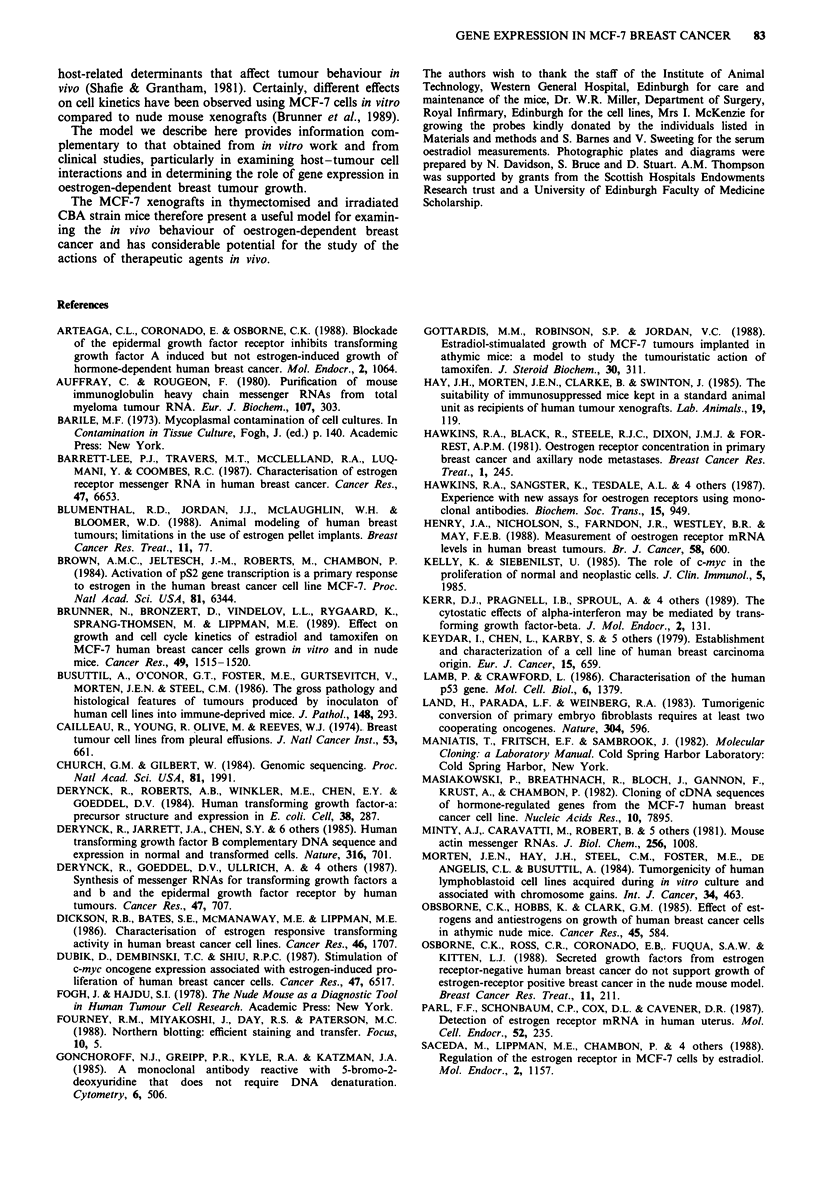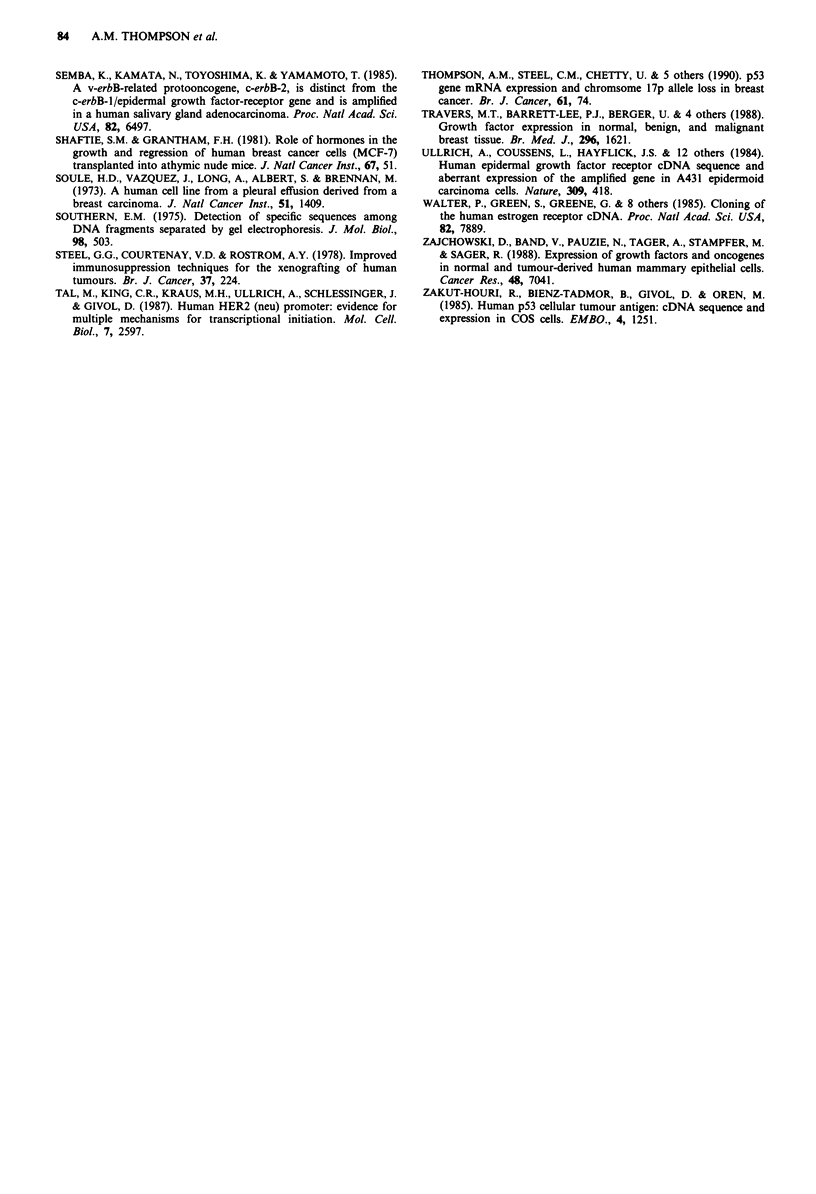# Gene expression in oestrogen-dependent human breast cancer xenograft tumours.

**DOI:** 10.1038/bjc.1990.233

**Published:** 1990-07

**Authors:** A. M. Thompson, C. M. Steel, M. E. Foster, D. Kerr, D. Paterson, D. Deane, R. A. Hawkins, D. C. Carter, H. J. Evans

**Affiliations:** Department of Surgery, Royal Infirmary of Edinburgh, UK.

## Abstract

**Images:**


Br. J. Cancer (1990), 62, 78-84 © Macmillan Press Ltd., 1990

### Gene expression in oestrogen-dependent human breast cancer xenograft tumours

A.M. Thompson[1,2], C.M. Steel[2], M.E. Foster[2], D. Kerr[3], D. Paterson[4], D. Deane[2], R.A. Hawkins[1], D.C. Carter[1] & H.J. Evans[2]

[1]*Department of Surgery, Royal Infirmary of Edinburgh, Lauriston Place, Edinburgh EH3 9YW;* [2]*MRC Human Genetics Unit, Western General Hospital, Crewe Road, Edinburgh EH4 2XU;* [3]*Beatson Institute, Garscube Estate, Switchback Road, Glasgow G61 1BD; and* [4]*Department of Pathology, University of Edinburgh, Teviot Place, Edinburgh EH1 1LS, UK.*

**Summary** Xenograft tumours from an oestrogen-dependent human breast cancer cell line MCF-7 have been established and characterised in thymectomised, irradiated female CBA strain mice. There was evidence for selection in xenografts of a subpopulation of MCF-7 cells with an altered pattern of gene expression as measured by mRNA levels compared with the original cells *in vitro*. Tumorigenicity increased significantly on repeated animal passage but oestrogen dependence was retained. Following injection of the mice with oestrogen, mitosis was induced in the tumour cells with associated increases in thymidine uptake and percentage of cells in S-phase. In accord with these changes, c-*myc* and p53 expression were increased and TGF-beta was suppressed. Thereafter the expression of the c-*myc* and p53 genes fell whilst that of the TGF-beta gene was induced as the oestrogenic stimulus declined. The oestrogen-regulated mRNA pS2 showed a biphasic response to oestrogen and levels declined as the serum oestrogen fell to undetectable levels. This xenograft system demonstrates that changes in transcription of oncogenes, growth factor and oestrogen-regulated genes can be detected *in vivo* in response to oestrogen. It thus provides an *in vivo* model for studies of the biochemical and molecular basis for therapeutic manipulation of hormone-sensitive human breast cancer.

In studying the molecular biology of cancer cells, the significance of *in vitro* observations may be uncertain due to the absence of host factors that influence tumour behaviour *in vivo*. Recent work on experimental human breast tumours *in vivo* has made use, almost exclusively, of congenitally athymic ('nude') mice (Osborne *et al.*, 1988; Brunner *et al.*, 1989). We have previously reported the growth of a range of human tumours in thymectomised, irradiated mice (Busuttil *et al.*, 1986) which have advantages in ease of husbandry and cost (Steel *et al.*, 1978; Morten *et al.*, 1984; Hay *et al.*, 1985). We report here the characterization of an oestrogen-responsive tumour derived from the MCF-7 human breast carcinoma cell line grown *in vivo* in female thymectomised and irradiated CBA mice. In addition, we have examined the kinetics of the expression of a range of related genes (c-*myc*, p53, TGF-beta and pS2) following oestrogenic stimulation in this tumour model system.

### Materials and methods

Twenty-one day old female mice from an established breeding colony of CBA/Ca strain mice at the Institute of Animal Technology, Western General Hospital, Edinburgh, maintained as described in Hay *et al.* (1985), were anaesthetised with ether and suction thymectomy performed. Three weeks after thymectomy, 200 mg $kg^{-1}$ arabinoside C (Pfizer, UK) was injected by the intraperitoneal route and 48 h later the mice were irradiated to a total body dose of 7.50 Gy. Radiation was delivered from an X-ray source (250 kv: 0.3-0.4 Gy $min^{-1}$) with a Thoreus II filter. MCF-7 cells (Soule *et al.*, 1973) were cultured in Nunclon flasks (Nunc, Kamstrup, Denmark), fed regularly with Dulbecco's Modified Eagle Medium (DMEM; Gibco, Paisley, UK) containing phenol red (which has an oestrogenic effect on MCF-7 cells) supplemented with 12% fetal calf serum (FCS; Gibco) and maintained in an atmosphere containing 5% $CO_2$ at 37°C. All cultures were persistently negative for mycoplasma using conventional immunofluorescence techniques (Goding, 1983). The cells were harvested in the logarithmic (subconfluent) phase of growth and washed twice in phosphate-buffered saline (PBS). A single dose of $10^7$ viable cells suspended in 50-100 µl PBS was injected into the fourth right mammary fat pad 1-3 weeks after irradiation. At this time 50 µg oestradiol benzoate (Intervet UK Ltd., Cambridge, UK) in 50 µl arachis oil was injected subcutaneously into the nape of the neck. This injection of 50 µg oestradiol benzoate in arachis oil was repeated every three weeks to the same site. MCF-7 cells were also injected into 20 CBA mice without oestrogen supplementation. A further 20 mice were tested with a second regime: either a 1.25 mg or a 0.5 mg oestradiol 6-week-release pellet (Innovative Research, Ohio, USA) was placed subcutaneously. Tumours were measured daily in two dimensions by the same author using calipers. The volume of the tumour was calculated using the formula pi/12 × (mean diameter)$^3$.

Mice were killed at selected times and the tumours were frozen immediately in liquid nitrogen or fixed for histology. Those mice which did not develop tumours were killed 90 days from the start of the experiment. Mice which died in the interim were examined in detail for evidence of disease.

Fragments of xenograft material arising from inocula of MCF-7 cells were further transplanted through a dorsal incision into additional thymectomised, irradiated mice under anaesthetic. An injection of 50 µg 17 beta oestradiol benzoate in arachis oil was administered as the mouse awoke. Thereafter the transplant-bearing mice were treated as above. Remaining tumour fragments were macerated and added to DMEM containing 12% FCS and the tumour cells returned to culture for 8 weeks before reinjection into mice or extraction of total ribonucleic acid (RNA).

Two additional human breast cancer cell lines MDA-MB-231 (Cailleau *et al.*, 1974) and T47-D (Keydar *et al.*, 1979) were cultured and similarly maintained. The cells were harvested in the logarithmic phase of growth so that the RNA could be extracted for comparison with that from the MCF-7 cells.

### *Serum oestradiol concentration*

Serum was prepared from individual samples of blood (0.3-0.8 ml) taken by cardiac puncture from each of 30 mice at specified times following the 3-weekly oestradiol benzoate injections. The concentration of 17 β-oestradiol was determined in 50 µl samples of serum using a commercially available radioimmunoassay (Baxter Health Care, Newbury,

Correspondence: A.M. Thompson[1].
Received 29 November 1989; and in revised form 28 February 1990.

Berks., UK). Within assay CV was 5% and between assay CV was 6%.

*Cellular DNA synthesis*

*Percentage of cells in 'S' phase.* A monoclonal antibody, BR9, directed against the halogenated nucleotide 5-bromodeoxyuridine (5BrdU) was raised by one of the authors (D.D.) and used for rapid S-phase measurements (Gonochoroff *et al.*, 1985).

A total of 200 μl of 6 mg $ml^{-1}$ 5-BrdU (Sigma) was injected into the peritoneal cavity of each mouse under examination at 0, 6, 12, 18, 24, 36, or 48 h after injection of 50 μg 17 β-oestradiol. Each mouse was killed 1 h after administration of the BrdU and the cells were released from the tumours by dispase digestion and fixed in 70% ethanol. A double staining procedure was used for the differentiation of human MCF-7 cells from invading host (mouse) cells. Fixed cells were incubated with 10 μg of an antihuman class I monoclonal antibody PE25 (D.D.), washed in PBS and 2 μg of phycoerythrin-labelled anti-mouse conjugate added (Southern Biotechnology Associates Inc.). To prevent cross-reactivity of this antibody with the BR9, the cells were then incubated in PBS containing 10 μg $ml^{-1}$ mouse IgG (Sigma) for 30 min. After a wash in PBS, 10 μg of the monoclonal antibody BR9 was added to the pellet of $10^6$ cells and incubated at room temperature for 40 minutes, washed in PBS and incubated for a further 40 minutes in the presence of 2 μg fluoroscein isothiocyanate (FITC)-labelled goat anti-mouse conjugate (Sigma). After a final wash in PBS the cells were analysed on a FACScan flow cytometer (Becton Dickson, Lincoln Park, NJ, USA) for the absolute number of cells and the proportion of tumour cells in S phase.

*Thymidine incorporation* Thymidine uptake was also used as an index of DNA synthesis in the isolated tumour cells. For each time point, intact cells were separated from the dispase/DMEM digests by density centrifugation over 5–30% Ficoll 400 gradient (Pharmacia, Uppsala, Sweden) and 150 μl of cell suspension ($10^6$ cells $ml^{-1}$ DMEM with 5% added FCS) were added per well of a 96-well mitcrotitre plate (Falcon 3072, Becton Dickson). One μCi $^3$H-thymidine (Amersham International, Aylesbury, UK) was added to each well and the cells were harvested 5 h later on to glass fibre discs. The radioactivity emitted from each disc was counted in 5 ml of scintillant (Opti-Scint, Pharmacia, Sweden) in a Packard 1600CA analyser (Packard, Downers Grove, Illinois, USA).

*Histopathology*

A slice through the middle of each tumour was fixed in methacarn for 1 h then 95% ethanol and then processed routinely. Paraffin sections were cut and examined after staining with haematoxylin and eosin. The mitotic index for each tumour was calculated from the mean number of mitoses in 50 randomly chosen, high-power fields (× 400) by one author (D.P.).

*Oestrogen receptor concentration*

The soluble oestrogen receptor concentration of xenograft tissue or cells was measured following homogenisation by a standard method (Hawkins *et al.*, 1981) and use of the Enzyme Immuno-Assay (EIA; Kit from Abbott Laboratories, North Chicago, Illinois). Both for the cells and for the solid tumours receptor concentration was expressed in fmol mg $proteins^{-1}$ (Hawkins *et al.*, 1987).

*Extraction of ribonucleic acid and northern blotting*

From frozen tumour, the total ribonucleic acid (RNA) was extracted using a modification of the method of Auffrey and Rougeon (1980). A known weight of frozen tumour was pulverised using a Mikrodismembrator II (Braun, FR Germany) and the resulting powder was finely disrupted using a plastic pipette in the presence of 2 ml per 100 mg tissue of 3M lithium chloride/6 M urea and left at 4°C overnight. Alternatively, cells cultured *in vitro* were washed in PBS and then disrupted in 3 M lithium chloride/6M urea with a plastic pipette. The DNA was sheared using a Soniprep 150 ultrasonic disintegrator (MSE Scientific Instruments, Crawley, UK) with an ice jacket. The RNA was recovered by centrifugation at 12,000 r.p.m. and the pellet was taken up in 6 ml 10 mmol Tris buffer pH 7.0, 0.1% sodium dodecyl sulphate (SDS), 300 μg proteinase K (Boehringer Mannheim, FR Germany) were added and the tube incubated at 37°C for 20 min. Protein was extracted using phenol equilibrated with 0.1 M Tris at pH 7 and 24:1 chloroform:isoamylalcohol. Following ethanol precipitation of the aqueous phase at −20°C, the RNA was recovered by centrifugation and dissolved in diethyl pyrocarbonate (DEPC, Sigma, USA) treated, autoclaved, distilled water and stored in aliquots at −70°C. The quantity and purity of the RNA was assessed spectrophotometrically at 260 nm and 240 nm. Throughout the RNA extraction procedures, sterile disposable plastic ware was used where possible; all solutions were made up with autoclaved DEPC-treated water, using baked glassware and gloves were worn to minimise exogenous ribonuclease contamination (Maniatis *et al.*, 1982).

Twenty micrograms of total RNA was denatured with formamide and formaldehyde at 55°C for 20 min; 2 μl loading buffer (50% glycerol, 1 mM EDTA, 0.4% bromophenol blue, 0.4% xylene cyanol) and 1 μl of 10 μg $\mu l^{-1}$ ethidium bromide were added to each sample. The denatured specimens were loaded on to a 1.1% agarose gel containing 0.66 M formaldehyde, submerged beneath MOPS buffer (0.2 M morpholinopropanesulphonic acid pH 7.0, 50 mM sodium acetate pH 7.0, 5 mM EDTA) and the RNA species were separated electrophoretically (modified from Fourney *et al.*, 1988).

The gel was washed in two changes of 10 × standard saline citrate (1 × SSC contains 150 mM sodium chloride, 15 mM sodium citrate, 1 mM EDTA, pH 7.4), photographed under a UV transilluminator and the RNA was transferred to a nylon filter (hybond-N, Amersham, UK) by hydrostatic action using 10 × SSC over 8 h (Southern, 1975). The hybond was rinsed in 2 × SSC, air-dried and the RNA was covalently fixed to the membrane using a UV transilluminator. The hybond and the remaining gel were photographed to check for adequate transfer of the RNA.

*Probe hybridisation*

Filters were prehybridised in 7% SDS, 0.5 M disodium hydrogen phosphate pH 7.2 and 1 mM EDTA pH 7.0 (modified from Church & Gilbert, 1984) for 30 min at 65°C. To this was added $^{32}$P cytidine triphosphate (CTP)-labelled cDNA probes labelled to 1 × $10^7$ c.p.m. $ml^{-1}$ using the randomprime DNA labelling system (Boehringer Mannheim, FR Germany). $^{32}$P-CTP incorporated probe was separated from unincorporated radionucleotide using a Sephadex column (Nick column, Pharmacia, UK) and denatured before addition to the hybridisation solution.

cDNA probe inserts digested from their respective plasmids were used to detect messenger RNA (mRNA) species for three oncogenes (*erb*B-2; p53; c-*myc*) three growth factors or their receptors (epidermal growth factor receptor; transforming growth factor-beta; transforming growth factor-alpha) and two hormone-related genes (OR3, pS2).

For c-*erb*B-2, the KpnI-XbaI fragment of lamda 107 was used (Semba *et al.*, 1985); for p53, the 2.1 kb php53Bam cDNA (Zakut-Houri *et al.*, 1985); and for c-*myc*, pSV-c-*myc*-1 for exons 2 and 3 (Land *et al.*, 1983). Epidermal growth factor receptor (EGFR) was detected with the 3.9 kb pHER-A64-1 probe (Ullrich *et al.*, 1984), transforming growth factor-alpha (TGF-alpha) with a 1.05 kb insert from Sp64-BC1 (Derynck *et al.*, 1984) and transforming growth factor beta with the 1.3 kb insert from Sp65-C17N (Derynck *et al.*, 1985). The two hormone-related probes were the 1.6 kb OR3 oestrogen receptor cDNA (Walter *et al.*, 1985) and the pS2

0.56 kb cDNA for oestrogen-regulated mRNA (Masiakowski *et al.*, 1982). As a standard probe, the Pst 1 insert cDNA of plasmid 91, detecting mouse alpha-actin mRNA specific sequences (Minty *et al.*, 1981), was used to quantify accurately each total RNA sample loaded. It is of particular relevance in this oestrogen-sensitive model that transcription of actin mRNA in MCF-7 cells is not affected by oestrogen (Saceda *et al.*, 1988).

Following hybridisation for 24 hours, filters were washed to remove non-specifically attached probe in two changes of 0.1% SDS 10 mmol disodium hydrogen phosphate wash buffer at 65°C with agitation. Filters were blotted dry, wrapped in cling film and exposed to preflashed Kodak XAR film at −70°C for up to 14 days. The extent of hybridisation of radiolabelled probe to the mRNA species was determined from densitometry using a laser densitometer constructed by the Medical Research Council Human Genetics Unit and expressed with respect to hybridisation to the actin probe. The size of mRNA species was calculated from the position of ribosomal RNA markers. Filters were reprobed up to six times with different cDNA probes; before reprobing, filters were stripped of residual probe by washing at 80°C for 60 min in 0.1% SDS and the filter was checked by autoradiography.

### Results

One hundred and forty-five CBA strain mice were injected with MCF-7 cells in this study (Table I). Of the 145 mice injected, 69 received both cultured MCF-7 cells and oestradiol benzoate. Twelve of these 69 mice died before 90 days leaving 57 for analysis, 30 of which (53%) grew a tumour. Of 36 mice into which tumour was transplanted, four died prematurely and 23/32 (72%) of the remaining mice grew tumours. The recultured cells grew as xenografts in 16 of the 18 mice injected which survived (89%). The take rate of transplanted tumour material was significantly higher than that of primary inocula ($P = 0.04$ by Fisher's exact test) and was higher still for tumour cells that had been recultured *in vitro* and then inoculated into fresh mice ($P = 0.004$ by Fisher's exact test).

Tumours did not grow without oestrogen supplementation. The 50 µg oestradiol benzoate in arachis oil or the arachis oil alone were well tolerated by the CBA mice and the oestrogen delivered in this form promoted tumour growth. The use of conventional pellets of 1.25 mg or 0.5 mg oestradiol resulted in 20/20 deaths within 10 days. No consistent cause for these deaths was evident at *post mortem*.

### *Histopathology*

Each MCF-7 tumour was firm, pale, solid and well circumscribed, not showing overt local invasion or ulceration of the overlying skin. All the tumours were examined histologically and were compatible with an origin from breast, although they did not show marked adenocarcinomatous differentiaton. There were no areas of necrosis in the smaller tumours, although the larger tumours did have evidence of central necrosis. No marked lymphocyte infiltration was noted.

Microscopic and macroscopic examination of mice which died and mice which were killed to obtain tumour showed evidence of metastasis in only one animal. In that instance, tumour cells were evident at the site of MCF-7 cell inoculation, as peritoneal seedlings and microscopically in the visceral pleura of the lung. No pathological evidence of oestrogen toxicity was found at post mortem in any animal although there was some hair loss at the site of the oestradiol injection.

### *Serum oestradiol concentration*

Oestradiol was not detectable in the serum from thymectomised and irradiated mice prior to injection. After injection with 50 µg oestradiol benzoate (Figure 1), a sharp rise to a mean 7,492 pmol $l^{-1}$ (s.d. 3,374 pmol $l^{-1}$) oestradiol occurred by 24 h, declining exponentially to undetectable levels (less than 53 pmol $l^{-1}$) 2 weeks after the injection.

### *Tumour growth*

The tumours became palpable during the first 3 weeks following 17 β-oestradiol injection and following the second and third injections of oestrogen, the tumour was observed to grow in size, but not in a uniform fashion (Figure 1). In particular, during the first 14 days after injection, the tumour increased rapidly in size, then from day 14 to 21 slowed down or became static. By 3–6 weeks, all tumours were large enough for the studies described.

### *Cellular DNA synthesis and mitoses*

Increased thymidine uptake was noted by 18 h following the oestrogen injection (Figure 2) and, in parallel with the percentage of S-phase cells, reached a maximum 24 h following oestrogen injection, declining thereafter.

The number of mitoses per ×400 field (Figure 2) showed an increase, compared to the baseline value of 3 per ×400 field, to 24 per ×400 field demonstrable 24 h following oestrogen stimulation of the tumour. The level fell to 5 mitoses per ×400 field 10 days later. The number of mitoses showed parallel changes to the biochemical indices of cellular DNA synthesis.

**Figure 1** Serum oestradiol and tumour volume in a mouse xenograft model. Serum oestradiol (mean ± standard deviation) in thymectomised and irradiated mice for 2–4 mice at each time point following subcutaneous injection of 50 µg 17 β oestradiol benzoate. From undetectable levels (<53 pmol $l^{-1}$) prior to injection of oestradiol there is a rapid rise to a peak of 7,492 pmol$^{-1}$ followed by a decline in serum oestradiol to undetectable levels by the third week post-injection (solid dots). Tumour volume (calculated from pi/12 × mean diameter$^3$) for a cohort of 12 mice (mean volume ± standard deviation) measured during weeks 3 to 6 following injection of the MCF-7 cells. Only four time points are shown for clarity (open dots). There is a rise in tumour volume for the 2 weeks following injection of oestrogen, with little change in tumour volume once serum oestradiol becomes undetectable.

**Table I** The fate of MCF-7 cells inoculated into immunocompromised mice

| MCF-7 inoculum | Oestrogen supplement | Number of CBA mice | Number of live mice bearing tumours | Number of live mice without tumours | Deaths |
|---|---|---|---|---|---|
| Cells | Nil | 20 | Nil | 20 | Nil |
| Cells | 50 µg oestradiol benzoate | 69 | 30 (53%) | 27 | 12 |
| Transplant xenograft | 50 µg oestradiol benzoate | 36 | 23 (72%) | 9 | 4 |
| Recultured xenograft cells | 50 µg oestradiol benzoate | 20 | 16 (89%) | 2 | 2 |

**Figure 2** Three indices of cellular proliferation in xenografts of MCF-7 cells in mice following administration of oestradiol. Cell proliferation measured by $^{3}$H-dTr uptake (solid dots), percentage of cells in S phase (open dots) and mitoses per × 400 field (solid squares) showing mean value of four tumours for each time point. The same tumours were used for all three indexes of cell proliferation. Between 12 and 24 h following injection of 17 β oestradiol benzoate, MCF-7 cells are stimulated to divide, DNA synthesis returning to prestimulation levels by 48 h and mitoses declining within days.

*Oestrogen receptors*

MCF-7 cells *in vitro*, tumour material taken immediately before oestradiol injection and cells cultured from xenografts had a mean 120 fmol (range 110–135 fmol) oestrogen receptors per mg protein. There was a rise to 240 fmol mg$^{-1}$ protein at 30 h, but at 7, 14 and 21 days following injection, the level had returned to between 120 and 150 fmol mg$^{-1}$ protein.

*Tumour levels of mRNA*

*Presence of mRNA species* Messenger RNA was detected in MCF-7 cells by seven of the nine probes (Figure 3). No mRNA for EGFR or TGF-alpha was detected in the MCF-7 cells or xenografts, although both mRNA species were detected in other breast cancer cell lines (MDA-MB-231 and T47-D). While some mRNA species (c-*erb*B-2 and OR3) detected in the MCF-7 cell line were not seen in the xenograft material, no mRNA species was detected in the tumours which was not present in the original cell line (Figure 3). The mRNA for c-*erb*B-2 was detected at 3.0 kb and 1.8 kb in the original cell line but not in the xenografts.

**Figure 3** Gene expression in MCF-7 cells *in vitro* and *in vivo* (xenografts). Representative autoradiographs of MCF-7 cells, MCF-7 xenografts and MCF-7 cells re-cultured from the xenografts after probing with cDNA probes for c-*erb*B-2, p53, pS2, c-*myc* and TGF-beta (details in text). In each case the actin-probed control for each lane is shown. Two c-*erb*B-2 species are evident in the cell line but are not seen in the xenografts. Four p53 mRNA species are demonstrated in the cell line but only one in the xenografts and re-cultured cells. Similarly, three species detected with the pS2 probe are seen in the cell line but only one species in the xenografts and cells cultured from those xenografts. The c-*myc* and TGF-beta probes detect identical species in both the MCF-7 cell line and the xenograft tumours.

Four mRNA species for p53 (three at 2.8 kb, one at 1.8 kb) were identified in the original cell line but only a single mRNA for p53 was found in tumours or in re-cultured cells. Similarly, three mRNA species were detected with the pS2 probe in the original cell line but only the single 0.6 kb mRNA in the tumours. Other cDNA probes such as those for TGF-beta and c-*myc* detected only a single mRNA (2.5 kb in each case) present in both the cell line and xenografts. There were no differences in any mRNA species detected between xenografts, transplanted tumours and tumour cells recultured for periods of up to 56 days.

*Changes in gene expression after oestrogen stimulation (Figure 4).* Densitometry permitted detection of changes in the levels of mRNA for c-*myc*, p53, TGF-beta and pS2 with respect to alpha-actin mRNA, following stimulation of the xenograft by oestrogen. The mRNAs for c-*myc* and p53 both increased then fell back towards the unstimulated level within the first 24 h while that for TGF-beta was rapidly suppressed, rising only as the oestrogen stimulus declined after 1 week. pS2 expression showed a biphasic response with an initial increase to 24 h, then suppression for 12 h, a less substantial increase by 48 h and finally a decline as the expression of TGF-beta increased.

### Discussion

*General characteristics*

We have established the MCF-7 breast cancer cell line as xenografts in CBA mice immunocompromised by thymectomy and whole body irradiation. Tissue from these tumours can be transplanted to similar mice, and cells cultured from the xenografts can be re-implanted to grow tumours. This model therefore yields a large renewable supply of tumour material passaged *in vivo* and permits the study of tumours during hormonal manipulation. These MCF-7 tumours were clearly adenocarcinomata, with necrosis in only the larger tumours as in nude mice (Osborne, 1988) and metastasis, as previously noted, a rare event (Busuttil *et al.*, 1986).

As in nude mice, oestrogen supplementation is a prerequisite for MCF-7 tumour growth (Shafie & Grantham, 1981; Osborne *et al.*, 1985; Gottardis *et al.*, 1988). The absence of detectable serum oestrogen in female mice prior to injection confirmed that the mice had been 'oophorectomised'

**Figure 4** Changes in mRNA following oestrogenic stimulation of MCF-7 xenografts. Host animals were injected with 17β oestradiol benzoate at time 0. The changes shown are for c-*myc*, p53, pS2 and TGF-beta mRNA species in xenografts, as detected by densitometry of autoradiographs, with respect to that for actin as a control. The percentage at each time point is the mean of six tumours. mRNA expression for the same four species is shown for the original MCF-7 cells and cells recultured from xenograft tumours. Levels of c-*myc* (open squares) and p53 (solid dots) mRNA reach a peak within 12 h and decline to prestimulation levels by the second week. pS2 mRNA (solid squares) shows a biphasic response, with a peak at 12 h, apparent suppression of this peak to coincide with the peak serum oestradiol at 24 h and then a further substantial rise and then gentle decline to prestimulation values by the second week. In contrast to the other three species, TGF-beta is slightly suppressed in the first 24 h and peaks at day 7, returning gradually to prestimulation levels by day 21 (open dots).

by the irradiation. The serum oestrogen profile following a single subcutaneous injection of oestradiol benzoate in arachis oil (Figure 1) gave very high and possibly even inhibitory serum levels of oestradiol between 12 and 36 h. From day 2 to day 8, levels of oestradiol were within the physiological range for non-pregnant premenopausal women (110–1,630 pmol $l^{-1}$, data from Baxter Health Care, Newbury, UK) and they remained sufficient to sustain oestrogen-dependent tumour growth for 21 days. Serum oestradiol levels in surgically oophorectomised nude mice which have received the implantable pellet also fall within the same range (Shafie & Grantham, 1981; Blumenthal *et al*., 1988). We have no explanation for the intolerance of our experimental mice to the implantable pellet, although the findings reported here confirm our previous experience with this strain (M.E.F. and C.M.S., unpublished data).

DNA synthesis and mitotic activity of the tumour cells was evident in respone to oestradiol (Figure 2). The increase in mitoses from a mean of 3 per ×400 field to 24 per field is in keeping with observations in nude mice (5 and 25 mitoses per ×400 field respectively; Osborne *et al*., 1985). Similarly, despite possible influences on the thymidine pool by oestradiol, thymidine uptake and the % S phase cells confirmed the histological observation that, following oestrogen administration, there was an increase in cell proliferation, which preceded the increase in tumour volume.

The slight rise in oestrogen receptor protein following oestrogen stimulation of the tumour, from 120 fmol $mg^{-1}$ protein to 240 fmol $mg^{-1}$ protein, and its subsequent return to basal level is consistent with the view that oestrogen can stimulate the synthesis of its own receptor.

### *mRNA species*

The mRNA species detected, particularly in the MCF-7 cells *in vitro*, merit comparison with published data. Using the oncogene probes to study the RNA from MCF-7 grown *in vitro*, two distinct mRNAs of 1.8 and 3.0 kb were identified hybridising to the c-*erb*B-2 probe, both smaller than the single 4.8 kb mRNA species previously described (Semba *et al*., 1985) and identified in breast tumours and normal human tissues (data not shown). An amplified and rearranged epidermal growth factor receptor in epidermoid carcinoma cells generating a truncated 2.8 kb mRNA that encoded only the extracellular EGF binding domain has been reported previously (Ullrich *et al*., 1984). Just as the c-*erb*B-2 gene may be rearranged in the MCF-7 cells studied, truncated transcription may occur. Certainly, alternative transcription mechanisms have been proposed for the c-*erb*B-2 gene (Tal *et al*., 1987).

It is well recognised that a single gene can give rise to a variety of mRNA transcripts: the three p53 mRNA species of circa 2.8 kb similar to the 2.8 kb mRNA in human breast tumours (Thompson *et al*., 1990) may result from such mechanisms as different transcription initiation sites, differential splicing or other post-transcriptional modification. Certainly, the mean size observed corresponds well to published data for the human p53 mRNA (Zakut-Houri *et al*., 1985; Harlow *et al*., 1985), and the additional 1.8 kb mRNA identified is probably still large enough to encode a 53 kDa protein. The c-*myc* mRNA of 2.5 kb corresponds to that previously reported in MCF-7 cells (Zajchowski *et al*., 1988). The 2.5 kb mRNA for TGF-beta was of the expected size (Derynck *et al*., 1985; Travers *et al*., 1988).

Although mRNA for the epidermal growth factor receptor, and the mRNA for TGF-alpha, which acts upon it, have been reported in MCF-7 cells (Dickson *et al*., 1986, Arteaga *et al*., 1988), none was evident in the MCF-7 cell line tested here, suggesting that the MCF-7 cells in use in our laboratory may be variants of those used in some previous studies.

OR3, the probe for oestrogen receptor mRNA detected two messages of 3.0 and 1.7 kb, but no 6.2 kb mRNA. As in this study, Henry *et al*. (1988) were unable to demonstrate oestrogen receptor mRNA in the MCF-7 cell line using the OR3 cDNA clone, but demonstrated hybridisation to a 6.2 kb mRNA using their radionucleotide labelled RNA probe. While mRNA of 6.2 kb (Walter *et al*., 1985), 4.2 kb (Parl *et al*., 1987) and 3.7 kb (Barrett-Lee *et al*., 1987) has been reported in human tumour tissue and in the MCF-7 cell line, the species identified here do not correspond to any of these. It is therefore possible that the probe used did not detect the oestrogen receptor mRNA (perhaps due to the experimental conditions) or alternatively that the MCF-7 cells used in this study produce oestrogen receptor mRNA smaller in size than that previously identified. However, the translated oestrogen receptor protein was certainly present when assayed by enzyme immunoassay.

The pS2 probe appears to cross-hybridise to the same 3 kb and 1.7 kb mRNA as the OR3 probe but hybridises most strongly to a small mRNA of about 600 base pairs (Figure 3), corresponding to the oestrogen induced mRNA of Masiakowski *et al*. (1982). The biphasic change in pS2 suggests that the role of pS2 as a marker for oestrogen action may not be as simple as originally proposed.

### *mRNA differences in vitro and in vivo*

The original MCF-7 cells grown *in vitro* expressed several mRNA species not evident in the xenografts or in cells of these xenografts recultured for some time *in vitro*. These findings may indicate *in vivo* selection for a subpopulation of cells within the MCF7 culture.

The MCF-7 cells re-cultured from xenografts had an identical pattern of gene expression to the xenografts and did not, over an 8-week period, revert to the original MCF-7 cell line pattern. Serious consideration was given to the possibility that these findings could be due to a contaminant in the original cell line (such as mycoplasma) expressing the gene concerned or to contamination (perhaps by a plasmid) at some point in the RNA extraction or electrophoresis. Both these explanations are unlikely since tests on the MCF-7 culture (Barile, 1973) were persistently negative for mycoplasma, and no evidence of plasmid contamination was found in these or any other northern-blot RNA studies. Moreover, a range of different plasmids was used as vectors for the cDNA probes.

### *mRNA changes following oestrogen stimulation*

Oestrogen-induced stimulation of c-*myc* expression, previously noted in breast cancer cells *in vitro* (Dubik *et al*., 1987), was confirmed. The expression of c-*myc* and p53 at elevated levels in the xenograft tumours in response to oestrogen suggest that *in vivo* the expression of these two nuclear genes may be involved in cell cycling (Kelly & Seibenlist, 1985; Lamb & Crawford, 1986). *In vitro* work (Brown *et al*., 1984) identified an increase of pS2 mRNA in response to oestrogen which was attributed to increased transcription. The biphasic response of pS2 mRNA noted in this study may reflect initial oestrogenic stimulus then, as the oestrogen achieves a peak, inhibition of pS2 transcription, with subsequent pS2 stimulation as the serum oestrogen returns to more physiological levels.

Similarly, a decrease in TGF-beta transcription has been noted *in vitro* in response to oestrogen treatment of MCF-7 cells (Dickson *et al*., 1986). Both these effects were noted *in vivo* in response to oestrogen. In the present xenograft system, TGF-beta transcription increased as the mitogenic stimulus of oestrogen declined, compatible with the anti-proliferative effects noted on oestrogen receptor-positive breast cancer cell lines *in vitro* (Kerr *et al*., 1989).

### *Clinical implications*

Gene expression in the breast cancer cells, as detected by mRNA analysis, obviously changes when cells cultured *in vitro* grow as tumours *in vivo*. The physiological and clinical significance of *in vitro* observations have on occasion been controversial and may be difficult to interpret due to lack of host-related determinants that affect tumour behaviour *in vivo* (Shafie & Grantham, 1981). Certainly, different effects on cell kinetics have been observed using MCF-7 cells *in vitro* compared to nude mouse xenografts (Brunner *et al.*, 1989).

The model we describe here provides information complementary to that obtained from *in vitro* work and from clinical studies, particularly in examining host–tumour cell interactions and in determining the role of gene expression in oestrogen-dependent breast tumour growth.

The MCF-7 xenografts in thymectomised and irradiated CBA strain mice therefore present a useful model for examining the *in vivo* behaviour of oestrogen-dependent breast cancer and has considerable potential for the study of the actions of therapeutic agents *in vivo*.

The authors wish to thank the staff of the Institute of Animal Technology, Western General Hospital, Edinburgh for care and maintenance of the mice, Dr. W.R. Miller, Department of Surgery, Royal Infirmary, Edinburgh for the cell lines, Mrs I. McKenzie for growing the probes kindly donated by the individuals listed in Materials and methods and S. Barnes and V. Sweeting for the serum oestradiol measurements. Photographic plates and diagrams were prepared by N. Davidson, S. Bruce and D. Stuart. A.M. Thompson was supported by grants from the Scottish Hospitals Endowments Research trust and a University of Edinburgh Faculty of Medicine Scholarship.

### References

ARTEAGA, C.L., CORONADO, E. & OSBORNE, C.K. (1988). Blockade of the epidermal growth factor receptor inhibits transforming growth factor A induced but not estrogen-induced growth of hormone-dependent human breast cancer. *Mol. Endocr.*, **2**, 1064.

AUFFRAY, C. & ROUGEON, F. (1980). Purification of mouse immunoglobulin heavy chain messenger RNAs from total myeloma tumour RNA. *Eur. J. Biochem.*, **107**, 303.

BARILE, M.F. (1973). Mycoplasmal contamination of cell cultures. In *Contamination in Tissue Culture*, Fogh, J. (ed.) p. 140. Academic Press: New York.

BARRETT-LEE, P.J., TRAVERS, M.T., MCCLELLAND, R.A., LUQMANI, Y. & COOMBES, R.C. (1987). Characterisation of estrogen receptor messenger RNA in human breast cancer. *Cancer Res.*, **47**, 6653.

BLUMENTHAL, R.D., JORDAN, J.J., MCLAUGHLIN, W.H. & BLOOMER, W.D. (1988). Animal modeling of human breast tumours; limitations in the use of estrogen pellet implants. *Breast Cancer Res. Treat.*, **11**, 77.

BROWN, A.M.C., JELTESCH, J.-M., ROBERTS, M., CHAMBON, P. (1984). Activation of pS2 gene transcription is a primary response to estrogen in the human breast cancer cell line MCF-7. *Proc. Natl Acad. Sci. USA*, **81**, 6344.

BRUNNER, N., BRONZERT, D., VINDELOV, L.L., RYGAARD, K., SPRANG-THOMSEN, M. & LIPPMAN, M.E. (1989). Effect on growth and cell cycle kinetics of estradiol and tamoxifen on MCF-7 human breast cancer cells grown *in vitro* and in nude mice. *Cancer Res.*, **49**, 1515–1520.

BUSUTTIL, A., O'CONOR, G.T., FOSTER, M.E., GURTSEVITCH, V., MORTEN, J.E.N. & STEEL, C.M. (1986). The gross pathology and histological features of tumours produced by inoculaton of human cell lines into immune-deprived mice. *J. Pathol.*, **148**, 293.

CAILLEAU, R., YOUNG, R. OLIVE, M. & REEVES, W.J. (1974). Breast tumour cell lines from pleural effusions. *J. Natl Cancer Inst.*, **53**, 661.

CHURCH, G.M. & GILBERT, W. (1984). Genomic sequencing. *Proc. Natl Acad. Sci. USA*, **81**, 1991.

DERYNCK, R., ROBERTS, A.B., WINKLER, M.E., CHEN, E.Y. & GOEDDEL, D.V. (1984). Human transforming growth factor-a: precursor structure and expression in *E. coli*. *Cell*, **38**, 287.

DERYNCK, R., JARRETT, J.A., CHEN, S.Y. & 6 others (1985). Human transforming growth factor B complementary DNA sequence and expression in normal and transformed cells. *Nature*, **316**, 701.

DERYNCK, R., GOEDDEL, D.V., ULLRICH, A. & 4 others (1987). Synthesis of messenger RNAs for transforming growth factors a and b and the epidermal growth factor receptor by human tumours. *Cancer Res.*, **47**, 707.

DICKSON, R.B., BATES, S.E., MCMANAWAY, M.E. & LIPPMAN, M.E. (1986). Characterisation of estrogen responsive transforming activity in human breast cancer cell lines. *Cancer Res.*, **46**, 1707.

DUBIK, D., DEMBINSKI, T.C. & SHIU, R.P.C. (1987). Stimulation of c-*myc* oncogene expression associated with estrogen-induced proliferation of human breast cancer cells. *Cancer Res.*, **47**, 6517.

FOGH, J. & HAJDU, S.I. (1978). *The Nude Mouse as a Diagnostic Tool in Human Tumour Cell Research*. Academic Press: New York.

FOURNEY, R.M., MIYAKOSHI, J., DAY, R.S. & PATERSON, M.C. (1988). Northern blotting: efficient staining and transfer. *Focus*, **10**, 5.

GONCHOROFF, N.J., GREIPP, P.R., KYLE, R.A. & KATZMAN, J.A. (1985). A monoclonal antibody reactive with 5-bromo-2-deoxyuridine that does not require DNA denaturation. *Cytometry*, **6**, 506.

GOTTARDIS, M.M., ROBINSON, S.P. & JORDAN, V.C. (1988). Estradiol-stimualated growth of MCF-7 tumours implanted in athymic mice: a model to study the tumouristatic action of tamoxifen. *J. Steroid Biochem.*, **30**, 311.

HAY, J.H., MORTEN, J.E.N., CLARKE, B. & SWINTON, J. (1985). The suitability of immunosuppressed mice kept in a standard animal unit as recipients of human tumour xenografts. *Lab. Animals.*, **19**, 119.

HAWKINS, R.A., BLACK, R., STEELE, R.J.C., DIXON, J.M.J. & FORREST, A.P.M. (1981). Oestrogen receptor concentration in primary breast cancer and axillary node metastases. *Breast Cancer Res. Treat.*, **1**, 245.

HAWKINS, R.A., SANGSTER, K., TESDALE, A.L. & 4 others (1987). Experience with new assays for oestrogen receptors using monoclonal antibodies. *Biochem. Soc. Trans.*, **15**, 949.

HENRY, J.A., NICHOLSON, S., FARNDON, J.R., WESTLEY, B.R. & MAY, F.E.B. (1988). Measurement of oestrogen receptor mRNA levels in human breast tumours. *Br. J. Cancer*, **58**, 600.

KELLY, K. & SIEBENILST, U. (1985). The role of c-*myc* in the proliferation of normal and neoplastic cells. *J. Clin. Immunol.*, **5**, 1985.

KERR, D.J., PRAGNELL, I.B., SPROUL, A. & 4 others (1989). The cytostatic effects of alpha-interferon may be mediated by transforming growth factor-beta. *J. Mol. Endocr.*, **2**, 131.

KEYDAR, I., CHEN, L., KARBY, S. & 5 others (1979). Establishment and characterization of a cell line of human breast carcinoma origin. *Eur. J. Cancer*, **15**, 659.

LAMB, P. & CRAWFORD, L. (1986). Characterisation of the human p53 gene. *Mol. Cell. Biol.*, **6**, 1379.

LAND, H., PARADA, L.F. & WEINBERG, R.A. (1983). Tumorigenic conversion of primary embryo fibroblasts requires at least two cooperating oncogenes. *Nature*, **304**, 596.

MANIATIS, T., FRITSCH, E.F. & SAMBROOK, J. (1982). *Molecular Cloning: a Laboratory Manual*. Cold Spring Harbor Laboratory: Cold Spring Harbor, New York.

MASIAKOWSKI, P., BREATHNACH, R., BLOCH, J., GANNON, F., KRUST, A., & CHAMBON, P. (1982). Cloning of cDNA sequences of hormone-regulated genes from the MCF-7 human breast cancer cell line. *Nucleic Acids Res.*, **10**, 7895.

MINTY, A.J,. CARAVATTI, M., ROBERT, B. & 5 others (1981). Mouse actin messenger RNAs. *J. Biol. Chem.*, **256**, 1008.

MORTEN, J.E.N., HAY, J.H., STEEL, C.M., FOSTER, M.E., DE ANGELIS, C.L. & BUSUTTIL, A. (1984). Tumorgenicity of human lymphoblastoid cell lines acquired during *in vitro* culture and associated with chromosome gains. *Int. J. Cancer*, **34**, 463.

OBSBORNE, C.K., HOBBS, K. & CLARK, G.M. (1985). Effect of estrogens and antiestrogens on growth of human breast cancer cells in athymic nude mice. *Cancer Res.*, **45**, 584.

OSBORNE, C.K., ROSS, C.R., CORONADO, E.B,. FUQUA, S.A.W. & KITTEN, L.J. (1988). Secreted growth factors from estrogen receptor-negative human breast cancer do not support growth of estrogen-receptor positive breast cancer in the nude mouse model. *Breast Cancer Res. Treat.*, **11**, 211.

PARL, F.F., SCHONBAUM, C.P., COX, D.L. & CAVENER, D.R. (1987). Detection of estrogen receptor mRNA in human uterus. *Mol. Cell. Endocr.*, **52**, 235.

SACEDA, M., LIPPMAN, M.E., CHAMBON, P. & 4 others (1988). Regulation of the estrogen receptor in MCF-7 cells by estradiol. *Mol. Endocr.*, **2**, 1157.

SEMBA, K., KAMATA, N., TOYOSHIMA, K. & YAMAMOTO, T. (1985). A v-*erb*B-related protooncogene, c-*erb*B-2, is distinct from the c-*erb*B-1/epidermal growth factor-receptor gene and is amplified in a human salivary gland adenocarcinoma. *Proc. Natl Acad. Sci. USA*, **82**, 6497.

SHAFTIE, S.M. & GRANTHAM, F.H. (1981). Role of hormones in the growth and regression of human breast cancer cells (MCF-7) transplanted into athymic nude mice. *J. Natl Cancer Inst.*, **67**, 51.

SOULE, H.D., VAZQUEZ, J., LONG, A., ALBERT, S. & BRENNAN, M. (1973). A human cell line from a pleural effusion derived from a breast carcinoma. *J. Natl Cancer Inst.*, **51**, 1409.

SOUTHERN, E.M. (1975). Detection of specific sequences among DNA fragments separated by gel electrophoresis. *J. Mol. Biol.*, **98**, 503.

STEEL, G.G., COURTENAY, V.D. & ROSTROM, A.Y. (1978). Improved immunosuppression techniques for the xenografting of human tumours. *Br. J. Cancer*, **37**, 224.

TAL, M., KING, C.R., KRAUS, M.H., ULLRICH, A., SCHLESSINGER, J. & GIVOL, D. (1987). Human HER2 (neu) promoter: evidence for multiple mechanisms for transcriptional initiation. *Mol. Cell. Biol.*, **7**, 2597.

THOMPSON, A.M., STEEL, C.M., CHETTY, U. & 5 others (1990). p53 gene mRNA expression and chromsome 17p allele loss in breast cancer. *Br. J. Cancer*, **61**, 74.

TRAVERS, M.T., BARRETT-LEE, P.J., BERGER, U. & 4 others (1988). Growth factor expression in normal, benign, and malignant breast tissue. *Br. Med. J.*, **296**, 1621.

ULLRICH, A., COUSSENS, L., HAYFLICK, J.S. & 12 others (1984). Human epidermal growth factor receptor cDNA sequence and aberrant expression of the amplified gene in A431 epidermoid carcinoma cells. *Nature*, **309**, 418.

WALTER, P., GREEN, S., GREENE, G. & 8 others (1985). Cloning of the human estrogen receptor cDNA. *Proc. Natl Acad. Sci. USA*, **82**, 7889.

ZAJCHOWSKI, D., BAND, V., PAUZIE, N., TAGER, A., STAMPFER, M. & SAGER, R. (1988). Expression of growth factors and oncogenes in normal and tumour-derived human mammary epithelial cells. *Cancer Res.*, **48**, 7041.

ZAKUT-HOURI, R., BIENZ-TADMOR, B., GIVOL, D. & OREN, M. (1985). Human p53 cellular tumour antigen: cDNA sequence and expression in COS cells. *EMBO.*, **4**, 1251.